# A review of the discussions on cultivated meat from the Islamic perspective

**DOI:** 10.1016/j.heliyon.2024.e28491

**Published:** 2024-04-02

**Authors:** Mohammad Naqib Hamdan, Rufaihah Abdul Jalil, Mohd Anuar Ramli, Nasiibah Ramli, Mohd Nor Adzhar Ibrahim, Muhamad Firdaus Ab Rahman, Hussein ‘Azeemi Abdullah Thaidi, Nur Najwa Hanani Abd Rahman

**Affiliations:** a Academy of Islamic Civilisation, Faculty of Social Sciences and Humanities, Universiti Teknologi Malaysia, Johor Bahru, Johor Darul Takzim, Skudai, 81310, Malaysia; b Centre of Research for Fiqh Science and Technology, Ibn Sina Institute for Scientific and Industrial Research, Universiti Teknologi Malaysia, Johor Bahru, Johor Darul Takzim, Skudai, 81310, Malaysia; c School of Applied Science, Temasek Polytechnic, 21 Tampines Ave 1, 529757, Singapore; d Stem Cell Core Lab Laboratory, Healthy Longevity Translational Programme, Yong Loo Lin School of Medicine, National University of Singapore, Singapore; e Academy of Islamic Studies, Universiti Malaya, 50603 Kuala Lumpur, Malaysia; f Faculty of Quranic and Sunnah, Universiti Sains Islam Malaysia, Bandar Baru Nilai, 71800 Nilai, Negeri Sembilan Darul Khusus, Malaysia; g Faculty of Syariah and Law, Universiti Sains Islam Malaysia, Bandar Baru Nilai, 71800 Nilai, Negeri Sembilan Darul Khusus, Malaysia

**Keywords:** Novel food, Muslim consumers, Halal diet, Islamic perspective, Al-maqasid al-shariah, Cultivated meat, Islamic jurisprudence

## Abstract

Since the introduction of the cultivated meat burger in 2013, numerous discussions have transpired between researchers and consumers, manifesting in various forms such as academic publications, opinions on personal websites and interactions on social platforms. For Muslim consumers, a primary concern revolves around the halal status of cultivated meat, given the pivotal role of adhering to a halal diet as a divine obligation in their lives. Therefore, this article seeks to systematically review the existing literature on cultivated meat from an Islamic perspective as articulated by Muslim scholars, whether these perspectives are issued by an established fatwa organisation or representations of personal views. The sources incorporated into this analysis span from academic publications, newspaper articles, fatwa bodies, personal websites and interviews. Employing thematic analysis, five principal themes were discerned within the discourse among Muslim scholars regarding cultivated meat: (i) the ontological status of cultivated meat, (ii) the status and source of stem cells used in the cultivation process, (iii) the theological implications of cultivated meat production on altering God's creation, (iv) foundational principles for halal cultivated meat production, and (v) the contemporary necessity of cultivated meat from the perspective of *al-maqasid al-shariah*. It is duly recommended that international fatwa organisations such as the International Islamic Fiqh Academy (IIFA) engage in discussions and deliberations on this matter of growing significance within the food industry. The fatwas and resolutions issued by IIFA are frequently cited as the most recognised authority in many Islamic countries.

## 1 Introduction

Cultivated meat, also known as lab-grown meat or cell-based meat, is the outcome of *in vitro* cultivation of animal cells in the laboratory. This innovative approach to meat production is a form of cellular agriculture where animal muscle tissue is grown in a controlled environment using animal stem cells, which are subsequently harvested and processed to create meat products. Despite being introduced in 2013, there are still numerous questions and debates about the viability and potential of the cultivated meat industry [1]. Notwithstanding the ongoing discussions, cultivated meat holds substantial potential and could emerge as an important source of meat for people in the future. In comparison to conventional meat production, one of the biggest advantages of cultivated meat is its potential to reduce the environmental impact of meat production [2,3].

Cultivated meat production boasts several environmental advantages, notably its reduced demand for land and water. This is achieved through its capability to produce meat in controlled laboratory settings or bioreactors, directly utilising animal cells [4]. These characteristics suggest that cultivated meat may substantially reduce the environmental footprint associated with conventional meat production, while also offering more sustainable and healthier sources of protein and enhanced hygienic standards [1,5]. Despite the advantages, the cultivated meat industry still faces some obstacles. Key challenges include the development of cost-effective, resource-efficient methods that can be scaled for mass production. Technical hurdles encompass aspects such as cell resources, proliferation and differentiation methods, serum-free media, bioreactor design optimisation tailored to various production stages, direct perfusion edible scaffolds, suspension culture techniques, microcarriers, innovative nutrient formulations, synthetic biology applications, and the custom production of artificial meat using 3D printing technology [6]. Another significant challenge facing the cultivated meat industry pertains to ensuring its compliance with halal dietary principles. Numerous studies have explored and are currently exploring consumer acceptance of cultivated meat, with particular emphasis on the crucial requirement of halal status for Muslim consumers. In light of this, the present article conducts an extensive examination of the discussions and perspectives put forth by Muslim experts regarding cultivated meat. This analysis encompasses multiple dimensions, including the conceptual framework of cultivated meat, the halal requirements integral to its production, and the characteristics of the final product. These perspectives are sourced from both established fatwa organisations and individual opinion pieces. The central motivation for this inquiry stems from the absence of a unified consensus on the halal status of cultivated meat, underscoring the necessity of addressing the factors contributing to these divergent viewpoints.

## 2 Halal diet and cultivated meat

The halal status of cultivated meat holds significant importance for Muslim consumers, as adherence to a halal diet represents a sacred duty ordained by God. A halal diet encompasses foods and drinks that are deemed permissible under Islamic law. Within this dietary framework, halal foods include plant foods, dairy products, eggs, poultry, and meat from animals slaughtered according to the Islamic ritual. However, it is imperative to note that alcohol is unequivocally prohibited within a halal diet.

In the context of halal diet, Muslims consumers embrace the *tayyib* concept as an integral component. The adoption of halal and *tayyib* dietary requirements finds its foundation in the teachings delineated in the Qur'an and *hadith* (a narrative record of Prophet Muhammad's sayings and actions). The term “halal” embodies the notion of lawful, permissible, or permitted within the framework of Islamic dietary laws. It refers to food, beverages, and other consumables that conform to the stipulated Islamic guidelines, thereby deeming them lawful for Muslim consumption. Fundamental tenets under ‘halal’ include the unequivocal prohibition of alcohol, no pork, no cross-contamination between halal and non-halal items, as well as the meticulous observance of Islamic teachings during the process of slaughter.

This comprehensive approach is paralleled by the “*tayyib*” concept, which transcends the mere permissibility associated with “halal.” “*Tayyib*” places an emphasis on the quality, wholesomeness, purity, and overall impact on health and well-being of food items. Foundational principles guiding ‘*tayyib*’ considerations encompass quality, safety, health and cleanliness. For example, a chicken that adheres to Islamic slaughtering guidelines, is properly cooked, and is prepared in a hygienic environment exemplifies the convergence of the halal diet and the *tayyib* concept, commonly referred to as *halalan tayyiban*. However, should the same chicken be prepared in a dirty and unsanitary environment, it may still be considered halal but would not meet the stringent criteria of the *tayyib* concept [[7], [8], [9]].

There are four places in the Qur'an where this issue is mentioned, namely (i) Surah al-Baqarah, chapter 2, verse 168, (ii) Surah al-Maidah, chapter 5, verse 5 (iii) Surah al-Anfal, chapter 8, verse 69 and (iv) al-Nahl, chapter 16, verse 114 (Quran, 2022). Below are the translations of the verses:

“O people, eat what is good and lawful (*halalan tayyiban*) from the earth, and do not follow Satan’s footsteps, he is your sworn enemy” (al-Baqarah, 2:168)

“This day, all good things have been made lawful for you” (al-Maidah, 5:5)

“So, eat the lawful and pure things (*halalan tayyiban*), and fear Allah. Surely, Allah is the Most-Forgiving and Very-Merciful” (al-Anfal, 8:69)

“So eat of the good and lawful things Gods has provided foryou and be thankful for His blessings” (al-Nahl, 16:114).

According to al-Qurtubi, in his commentary on verse 5, Surah al-Maidah, chapter 5, there exists a preceding verse with a significant relationship and continuity. He wrote: “The Companions of the Prophet asked him: What is lawful for them (Muslims)? Said the Prophet: All good things are lawful for you” (al-Maidah, 5:4) [7]. From these verses, Muslim scholars have derived an Islamic maxim or principle: “Any food is allowed to be consumed unless proven otherwise” [10]. This principle includes drinks, water, fruits, vegetables, seafood, nuts, milk, rice, wheat, eggs and more. But when it pertains to animals that need to be slaughtered, especially farm animals, the foundational rule is: “It is forbidden unless it is proven that the animal was slaughtered according to Islamic teachings” [11]. Al-Tabari expounds on this in his commentary: “O people, eat of the livestock which God has provided for you, which He has made lawful, permissible, good and slaughtered for you, and which is not forbidden to you”." He began by providing overarching principles and progressively refines them when applied to livestock [12]. Hence, if a Muslim desires to consume meat from a cow, lamb or chicken, he must ensure that the meat originates from animals slaughtered in accordance with Islamic teachings, which emphasise the importance of a proper slaughtering process [[13], [14], [15], [16], [17], [18], [19], [20], [21]].

This same criterion extends to halal cultivated meat, which must comply with *halalan tayyiban* dietary regulations and requirements. In a broader context, there are three main requirements concerning the production phase of cultivated meat, i.e. before, during and after the process. Before the production process, the source of the stem cells must be obtained from a halal animal such as lamb, cow, goat and chicken that has been slaughtered according to Islamic teachings. However, if the stem cells were obtained from marine animals, there is no requirement for slaughtering, and they can even be acquired while the animal is still alive. During the production process, the culture media, scaffolds, enzymes, growth factors and nutrients used are sourced from halal origins. The inclusion of serum may result in meat that is not halal. The producer must maintain strict hygiene standards and prevent cross-contamination with non-halal stem cells and substances during the production process. Lastly, the cultivated meat product must also be safe and not contain misleading information and labels [[22], [23], [24], [25], [26]].

## 3 Methods

For this review, an unrestricted comprehensive search of international databases was conducted until 3 March 2023. The databases searched included Web of Science (WOS), Science Direct, PubMed, Scopus, Google and Google Scholar. The search strategy was carried out using various terms and keywords, such as: *halal* (حلال), Islam (إسلام), Islamic perspective (المنظور الإسلامي), *fiqh* (فقه), *maqasid* (مقاصد), *fatwa* (فتوى), *mufti* (مفتي), Islamic law (الفقه الإسلامي) and cultured meat (or) cultivated meat (or) lab meat (or) *in vitro* meat (or) *daging kultur* (or) *daging makmal* (or) اللحوم الاصطناعي (or) اللحوم المعمالي. The reference lists of eligible articles were also scrutinised to identify similar studies. It should be noted that the selected references include individual opinion pieces, interviews with Muslim scholars, a newpaper article, research articles and official fatwas or statements in English, Arabic and Bahasa Melayu.

A comprehensive search and selection of various keywords in several databases yielded all potential studies. The authors independently assessed all study titles to determine their eligibility. The eligible articles were retained after the authors reviewed their abstracts. The authors then conducted a comprehensive review of the full text of the articles to identify the required information. Once the studies were selected, the authors independently examined and reviewed the full texts of the articles multiple times to ensure the inclusion of pertinent studies. The research team then systematically extracted data from the articles through a rigorous filtering process. During the review of titles, abstracts, full texts and extracted data, any disagreements between authors were resolved through team discussions or through the adjudication of third-party specialists with expertise in halal research.

**Fig. 1:**
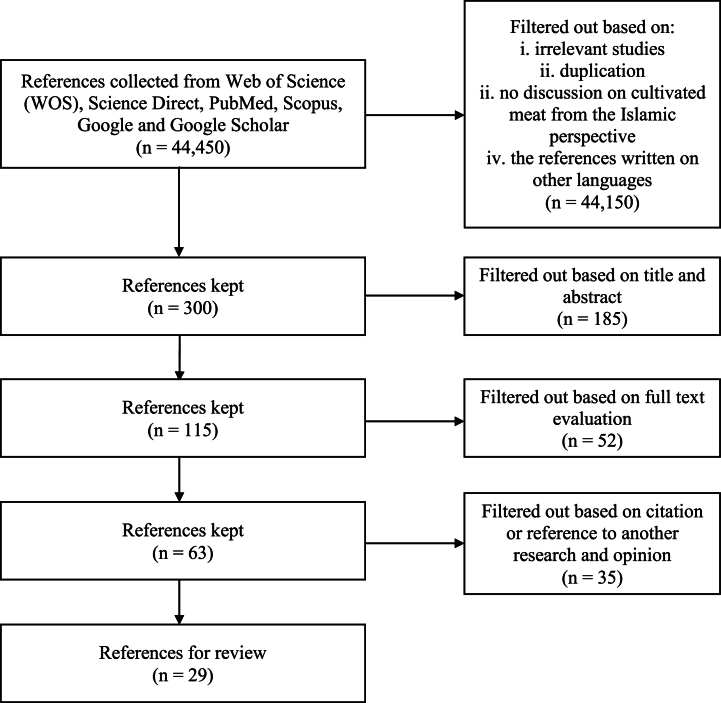
Process for identifying the relevant literature.

The initial search of the database found a total of 44,450 studies. Through the initial screening during the search and the exclusion of irrelevant studies, 300 studies were extracted for further analysis. After a meticulous review of the titles and abstracts, 115 texts were selected for in-depth investigation. The research team independently evaluated these texts independently to determine their relevance to the research topic. Subsequently, 52 texts were excluded from the study due to lack of information and relevance to the topic, however, 63 articles contained prospective features and therefore were retained. In accordance with the inclusion and exclusion criteria and the research objectives, 29 of the remaining 63 articles were included in the study. We filtered the references and sources according to the criteria as shown in Fig. 1 based on the inclusion and exclusion criteria in Table 1.

**Table 1:** Inclusion and exclusion criteria.

Inclusion criteria	Exclusion criteria
1. Present original data, not a citation to other opinions or research	1. Sources which cite or refer to another research or opinion
2. Focus on the discussion from an Islamic perspective	2. The period between 2013 and 2023
3. Language: Arabic, English and Bahasa	3. Review articles which do not include original opinion
4. Any type of sources including interviews, individual opinions, research articles, official fatwas and newspaper article	4. Other language than Arabic, English and Bahasa

**Table 2:** Research, opinions and interviews about cultivated meat.

Author(s), Reference	Type of References and affiliations	Title of the Article and language	Topics Covered and Conclusions
Billinghurst [27]	Interview with Muslim scholars (Dr. Abdul Qahir Qamar, Director of Fatwa and Sharia Rulings at International Islamic Fiqh Academy)	Is ‘shmeat’ the answer? In vitro meat could be the future of food	The ‘life’ of cultivated meat is vegetative and “similar to yogurt and fermented pickles” Halal cultivated meat consideration: i. Only cells from halal animal were used ii. No non-halal elements used during production, such as blood iii. No harm to human or environment
Kahf [28]	Live Fatwa on aboutislam.net by Dr. Monzer Kahf, Professor of Islamic Studies	Is Cultivated Meat, Made in a Lab, Halal or Haram?	He opined that taking a few cells in a syringe is different from cutting a portion of meat because it does not hurt or mutilate the animal
Hamdan [29]	Interview with Dr. Arif Ali Arif, Professor of Islamic Studies	The Use of Stem Cell in Culturing Meat: An Analysis from the Islamic Perspective	He claims that the source of stem cell is not a must to be obtained from slaughtered animal because the extraction didn't bring any hurt to animal
Hamdan and Ramli [30]	Research Article	Alteration of God's Creation Concept: An Analysis of Islamic Ruling on Cultivated meat Production	Cultivated meat research and production is not categorised as altering God's creation if it can bring benefits to humans and environment
Hamdan and Ramli [23]	Research Article	Cultivated meat in Islamic Perspective: An Analysis to the Use of ESCs as Source of Stem Cell	Halal cultivated meat must use stem cells extracted from slaughtered animals
Riaz and Arshad [31]	Research Article	Clean Meat, Opportunities, Challenges and its Halal Status	The process of cultivated meat production needs to be provided to Muslim scholars for halal determination
Musa Furber [32]	Individual Opinion	Comment: “A new lab-grown meat startup may have overcome a key barrier to making meat without slaughter”	The use of stem cells from non-slaughtered animals and blood serum is not allowed for halal cultivated meat production. He suggests other sources of stem cells from pregnant slaughtered animals, ie. umbilical cords and unborn animal babies.
Al-Munajjid [33]	Individual Opinion	Ruling on eating synthetic meat produced by using stem cells	He chooses to wait until clear information about cultivated meat can be achieved. His choice is known as *tawaqquf* in Islamic terminology
Hamdan et al. [24]	Research Article	Cultivated meat in Islamic Perspective	Halal cultivated meat production must start with the extraction of stem cells from a halal animal that was slaughtered according to Islamic teachings
American Fiqh Academy [34]	Fiqh Academy Opinion	Test Tube Turkey: Ruling on Meat Grown in Labs	Extracting muscle stem cells from live animals does not align with the Sharia law and results in non-halal cultivated meat. The use of impure medium such as fetal bovine serum during the process also led to the same ruling. But, extracting stem cells from chicken feathers using an induced pluripotent stem cell approach meets the Islamic guidelines for permissibility and does not require animal slaughter
Diaa [35]	Interview with Saad Jalloh, an Imam at Islamic Cultural Centre of New York	Is lab-grown meat halal? What experts say	A lot of things need to be discussed before formulating an opinion about its ruling
Purdy [36]	Interview with Aly Ghanim, the quality manager for the USA Halal Chamber of Commerce	Silicon Valley wrestles with religion. Is high-tech “clean meat” kosher and halal?	Part of the halal process is the animal has to be slaughtered properly
Hossain [37]	Research Article	Consumption of Stem Cell Meat: An Islamic Perspective	He discussed several points: i. Stem cells must be obtained from halal animals and slaughtered according to Shariah Law ii. The ‘life’ of cells in cultivated meat is not clear and it falls under the *syubhah* (grey area) condition. So, Muslims are advised to avoid it iii. No slaughtering and *tasmiyyah* (recite God's name) during the process needed after cultivated meat production is completed may result in a prohibited condition iv. The unnatural cultivated meat production may not align with the *tayyibah* concept in *halalan tayyiban* v. Cultivated meat production conspired as the violation of *sunnatullah* or natural law and the God has forbidden it in the Quran, Chapter 4, *Surah al-Nisa’*, verse 119. It also contradicted with the *al-maqasid al-shariah*
Gibril [38]	Interview with Dr. Samer al-Nass, an Islamic scholar from Syria	Cultured Chicken Meat	If no prohibited substance was used in its production, then there is no objection to consuming it
Awang [39]	Newspaper Article	Lab-produced food must pass religious, safety test	Cultivated meat is halal if the cells used originated from a halal-slaughtered animal, without the use of blood or animal-based serum, and/or growth enhancers in the manufacturing process. Cultivated meat must pass the safety test before entering the market
Wired [40]	Interview with Arzoo Ahmed, researcher at the Nuffied Council on Bioethics, UK	Halal or *haram*? For Islamic scholars, alt-meat is unfamiliar territory	Main question that needs to be discussed: is cultivated meat product is a meat or an animal product? Halal consideration for cultivated meat production: i. Approval stage of stem cell harvest ii. Stem cells harvested from halal animals slaughtered according to the Islamic ritual slaughter process iii. Culture medium and other products used in the cultivated meat production need to be halal
Reza Adnan et al. [9]	Research Article	Cultivated meat as *halalan tayyiban* food: A *maqasid* review in the preservation of life (*hifz al-nafs*)	Stem cells must be taken from halal slaughtered animals. Reza et al. also analysed cultivated meat consumption into three levels of necessity in preservation of human life, ie.: *daruriyyat* (the essential), *hajiyyat* (the complementary) and *tahsiniyyat* (the desirable or the embellishments)
Adam F [41]	Individual Opinion	Are synthetically produced burgers from stem cells halal?	Basic principles must be followed for halal cultivated meat production: i. Stem cells are extracted from an animal slaughtered in line with Islamic guidelines ii. Only halal ingredients/components are used iii. No cross-contamination with *haram* products
Mufti Ibrahim Desai [42]	Individual Opinion	What is the Islamic ruling on lab-grown meat?	Muscle stem cells are from among the part of an animal that has life within it. If the muscle stem cells were extracted without slaughtering the animal, it will be impure and impermissible. The use of fetal bovine serum (FBS) also may affect the halal status of cultivated meat. Any stem cell extracted from an animal's body that has not life, such as a feather, the cultivated meat that is produced by this type of stem cell is allowed even without slaughtering the animal
Hamdan, Post, et al. [25]	Research Article	Cultivated meat: Islamic and Other Religious Perspective	Several conclusions by the author: i. Cultivated meat production is not categorised as playing God or altering God's creation because it can fulfill human meat consumption and the results will benefit humanity in general ii. Stem cells must be extracted from halal animals iii. Stem cells from marine life can be extracted while the animal is still alive iv. No serum from blood used during the production process
Noordin [43]	Official fatwa by Mufti of Federal Territory's Office, Kuala Lumpur	Cultivated meat from Islamic perspective	Source of stem cells and their extraction will determine the halal status of cultivated meat product
Nahdatul Ulama Jawa Timur [44]	Official statement of Nahdlatul Ulama Institute of East Java province, Indonesia	Decision of the Regional Board of the Bahtsul Masail, Nahdlatul Ulama Institute of East Java concerning In Vitro Meat from Tissue Culture Results with Animal Product that has been Slaughtered	The Islamic verdict on cultivated meat production is described as follows: i. It is *haram* for cultivated meat consumption if the cells are obtained from animals that are still alive. The reason for this is that any cell, tissue, or body part that comes from a living animal is considered a carcass ii. It is halal if the cells that are cultured come from animals that are halal and have been slaughtered in accordance with Islamic teachings
Indiraphasa [45]	Official statement of Nahdlatul Ulama Institute of Indonesia	National Conference of Alim Ulama NU 2021 Decides Cell-Based Meat Consumption is Haram	NUI concludes that cell-based meat consumption is haram due to: i. Stem cells being extracted from living animals ii. Non-halal culture medium being used during the cultivated meat production process, ie. serum and gelatine iii. The conversion (*istihalah*) from non-halal substances to halal substance is not clear
Laila al-Shafie [46]	Interview with Dr. ‘Ajil al-Namshi, a Professor in Shariah and Islamic Studies	Cultivated Meat is Halal but With Several Requirements	The use of stem cells for food research and production is allowed according to the resolution by the International Islamic Fiqh Academy
	Dr. Bassam al-Shatti, Dr. Sa'd al-‘Anzi and Dr. Jalwi al-Jumai'ah, both Muslim scholars from Kuwait		The main requirement for halal cultivated meat production is that cell extraction originates from halal slaughtered animals and no non-halal substance is used during the production process
Wortley [47]	Interview with Dr. Mian Nadeem Riaz, Professor of Food Diversity	Global cultivated meat market set to boom, but is it halal?	If stem cells were derived from a slaughtered halal animal and the growth medium did not contain blood or serum, cultivated meat may be halal
Mohd Kashim et al. [48]	Research Article	Species-Specific Deoxyribonucleic Acid (DNA) Identification of Bovine in Cultivated Meat Serum for Halal Status	The use of fetal bovine serum (FBS) in cultivated meat production led to non-halal cultivated meat product. FBS is only used in laboratory research, but it will not be used as the main serum media in industrial production.
Han [49]	Interview with Abdul Majid, an Imam, and Islamic Slaughter consultant for UK-based halal certification organisation	Can lab-grown meat be kosher or halal?	If the animal was slaughtered properly and the meat used in the lab is for cultivated meat production, it is halal
Einhorn et al. [50]	Individual opinion from Mufti Taqi Usmani, an Islamic law expert	Can Lab-Grown Meat Really Be Halal or Kosher?	Permissible cultivated meat can only be produced if the original cells are extracted from slaughtered animals according to Shariah law
Izhar Ariff Mohd Kashim et al. [51]	Research Article	Scientific and Islamic perspectives in relation to the Halal status of cultivated meat	There are six principles suggested for halal cultivated meat production: 1. Only halal animals used for cell extraction and culture 2. The halal animal is slaughtered according to Islamic process 3. Not non-halal substances and elements used during the production process (ie. serum, biomaterial, scaffolding etc) 4. Perfect non-halal substance conversion from to a new substance form (*istihalah tammah*) 5. Consideration of *maslahah* (benefit) and *mafsadah* (damage) in cultivated meat production, with additional of al-maqasid al-shariah view (ie. preservation of life/soul, intellect, and property) 6. The needs level of cultivated meat still do not achieve *daruriyyat* (highest necessity) level

The studies consist of eight interviews, six individual opinion pieces, nine research articles, four official fatwas and one newspaper article (see Table 2). The 29 remainder articles underwent thematic analysis, hence revealed five primary themes reflecting the discussions and viewpoints of Muslim scholars concerning cultivated meat. These themes include (i) the characterisation of cultivated meat as it pertains to its ontological or ‘life’ aspects, (ii) the consideration of status and source of stem cells used in the culture process, (iii) the theological implications of cultivated meat production in relation to altering God's creation, (iv) the fundamental principles governing the for halal production cultivated meat and (v) the necessity of cultivated meat within the contemporary global context from the perspective of *al-maqasid al-shariah*.

## 4 Issues evolving from cultivated meat technology

### 4.1 First: the ‘life’ character of cultivated meat

In cultivated meat, its ‘life’ or its ontological aspect is a fundamental concern. The reproduction of cultivated meat from a single cell to produce a mass of edible meat shows its ‘life’ status. Classical Muslim scholars categorise life into two distinct forms: first, the life of humans and animals endowed with the ability to make choices and move independently, known as *hayah al-haiwan*. The second form of life, known as *hayah al-nabat*, is analogous to the reproductive and growth processes observed in plants and often referred as vegetative life [52]. These two life forms find expression in the human body itself. Human beings, as complete entities capable of independent movement, cognitive thought, and decision-making exemplify *hayah al-haiwan*. In contrast, the life of cells within the human body is beyond human control, such as the growth of hair and bones aligns with *hayah al-nabat* [53].

Al-Khudair also discusses this matter when elucidating the growth of hair and bones in animals. He posits that “living animals grow, as do living plants. The hair or fur of the animal that is elongated and ‘alive’ is in what kind of life, either *hayah al-haiwan* or *hayah al-nabat*? The more accurate situation is that it is *hayah al-nabat* (plant life), the same ‘life’ as a plant. So, the same is true of animal cells that reproduce until they become flesh; they are ‘alive’ but belong to the category of *hayah al-nabat*” [54]. Typically, *hayah al-haiwan* refers to the life of animals and humans as a complete organism, while *hayah al-nabat* refers to the life of plants and the life of cells or tissues in the bodies of animals and humans such as fur, hair and nails. Therefore, several contemporary Muslim scholars are of the opinion that cell multiplication to produce cultivated meat is similar to the concept of *hayah al-nabat* (vegetative life) [27,44,52]. But according to Hossain, the life status of cultivated meat is unclear, and it falls into a *syubhah* condition (grey area). Hence, muslims are advised to exercise caution and avoid it [37].

The status of cultivated meat and how it is categorised within the realm of Islamic dietary guidelines is a matter of significant discussion. A key question revolves around whether it should be classified as ‘meat’ or if it is more aptly described as an ‘animal product’ in a manner similar to eggs and cheese. The use of the word ‘*lahm*’ in the Qur'an and the sayings of the Prophet Muhammad suggests that it encompasses meat derived from various animals, including chicken, donkey, dog, pig, bird, and horse. Interestingly, even classical Muslim scholars such as *al-Sihah: Taj al-Lughah wa Sihah al-‘Arabiyyah* by Isma'il al-Jawhari (d.1003), *Lisan al-‘Arab* by Ibn Manzur (d.1233), *al-Misbah al-Munir* by Ahmad al-Fayyumi (d.1369) and *Taj al-‘Arus* by Muhammad Murtada (d.1790) do not list the word ‘meat’ in their dictionaries. This absence might be attributed to the Islamic doctrine that “whatever is known to people, need not to be defined” (*al-ma'ruf la yu'arraf*), as ‘meat’ is a term that has been understood by people for centuries [[55], [56], [57]].

There is an Islamic maxim that states that the Islamic judgement is determined by the analysis of the product and its production process rather than relying solely on the name or category (*al-‘ibrah bi al-musammayat, la bi al-asma’*). Hence, the categorization of the cultivated meat products, whether they are ‘meat’ or ‘animal product’, has no bearing on the Islamic evaluation of cultivated meat, but becomes significant when the product is ready for the consumer market. The name of the product holds considerable importance, as it can influence the consumer's decision to purchase the product. A noteworthy example is the renaming of the *Patagonian toothfish* as the *Chilean sea bass*, resulting in increased sales [58]. The term ‘conventional’ usually refers to food produced following standard practises. For example, conventional fruit or vegetables refers to produce that comes from the normal practise of using pesticides to control pests. Conventional meat refers to meat produced in the animal's body and processed after slaughter [59]. The Food and Agriculture Organisation of the United Nations (FAO), in its latest report on cell-based meat, also known as cultivated meat, distinguishes between conventional meat and cultivated meat and stressed the importance of product labelling [60] The name of the product carries significance, particularly when it has the potential to confuse consumers, leading them to believe that the cultivated meat product is a conventional meat product. Hence, this is not allowed because deception is forbidden in Islam according to the sayings of the Prophet Muhammad [61].

### 4.2 Second: the status and source of stem cells used in the cell culture process

The second topic of discussion is the classification of stem cells obtained from animal tissue. Are these stem cells considered similar to meat or is there a different, more appropriate categorization? This method, referred to as *qiyas*, involves equating a case that has not been explicitly stated in religious texts such as the Qur'an and the Prophet's sayings (hadith), with another case that has been clearly outlined, based on the similarities in the underlying causes present in both cases [62]. For example, the consumption of drugs such as cocaine and marijuana are not explicitly mentioned in the Qur'an or the Prophet's sayings but are considered prohibited in Islam due to its analogy with alcohol, which is clearly prohibited in religious texts.

While the production of cultivated meat is not mentioned in the Qur'an or the Prophetic Sayings, it is referenced in the hadith of Prophet Muhammad: “Whatever is cut off from an animal while it is still alive, then [the cut-off part] is dead (and will be carcass)” [63]. If the stem cells used for cultivated meat production are equated with the limbs of animals, as mentioned in this *hadith*, then it is necessary for the animal to be slaughtered first to ensure that the cultivated meat product is halal. This perspective is endorsed by most contemporary Muslim scholars [9,23,27,31,34,35,[39], [40], [41],^43^,^44^,^52^,^64^]. There is also a proposition of using stem cells from pregnant halal slaughtered animals, i.e. from the umbilical cord and unborn animal neonates with the condition that the animal must be slaughtered following halal procedures for stem cell collection [32].

However, some Muslim scholars take a different view, which is that equating stem cells with the limbs of animals is less accurate because extracting stem cells from an animal's body does not cause pain and does not mutilate the animal. Therefore, if this view is taken, then stem cell collection does not have to be performed on a slaughtered animal [28,29]. A third viewpoint is *tawaqquf*, which suggests that no definitive answer can be provided to questions without obtaining a clear understanding, in relation to the cultivated meat production process [33,35].

Another discussion revolves about the topic of reprogramming of stem cells from non-edible parts of animals such as hair follicles, skin, teeth or nails [34]. These stem cells are known as the induced pluripotent stem cells and they offer a potential source of cells used for cultivated meat. According to Islamic law, the fur of a permissible animal such as a cow or a chicken is not dirty or impure if it is taken alive or after slaughter. However, the fur of an animal carcass that has not been subjected to Islamic slaughter is deemed impure. Therefore, if scientists are able to obtain stem cells from animal hair follicles for cultivated meat production, it is imperative that the animal is either alive or has been slaughtered in accordance with Islamic teachings [[65], [66], [67]]. Thus, stem cells from the hair follicle of a halal living animal or after it has been slaughtered according to Islamic teachings are permissible for use in synthetic meat production.

Another source of stem cells that does not require the animal to be slaughtered is derived from blastocysts of fertilised eggs, which only apply to birds and chickens. The Israeli startup, Super Meat, has begun to use this technique for cultivated meat production on a larger scale. The bioreactor in the company's plant is currently 20,000 L and can produce several hundred kilograms of cultivated meat weekly [49]. In addition, they assert that this procedure is compatible with the kosher Jewish diet. Based on the Islamic perspective, Islamic scholars agree on the halal status of eggs.

According to the consensus of Islamic scholars, if a halal animal lays an egg while it is alive, after it has been slaughtered, or even after its death (i.e., it died without being slaughtered), the egg can be consumed as long as it is not spoiled. This is because eating animal eggs does not require the animal to be slaughtered [[14], [15], [16], [17], [18], [19], [20], [21],^68^]. However, if the egg has been fertilised and has developed into an embryo, which is considered “blood”, or has become a chick inside the egg, it is considered impure (*najis*). However, once it transforms into a chicken and hatches, it becomes pure and halal to be consumed [[14], [15], [16], [17], [18], [19], [20], [21],^69^].

Therefore, if cultivated meat is produced from embryos extracted from chicken and bird eggs, it would be considered pure and edible because its structure has shifted from being an embryo (considered blood) to chicken flesh or cultivated chicken. This transformation is known as *istihalah* (complete transformation), similar to the transformation of an embryo in an egg into a chicken. In contrast, extracting embryos from living animals, such as cows and goats, would require the animal to be slaughtered, which is different from extracting embryos from chicken or bird eggs.

### 4.3 Third: the production of cultivated meat is altering God's creation

A significant point of discussion revolves around whether the production of cultivated meat constitutes an alteration of God's creation and, consequently, whether its consumption should be prohibited for Muslims. In this context, natural law refers to the original production or creation of meat through animal farming and slaughter. This concept, also known among Muslim scholars as the “alteration of God's creation” or *taghyir khalqillah* in Arabic, argue that the new method of meat production represents an attempt to change the natural order of meat production. They supported their argument by referring to the Quranic verse in Surah al-Nisa’, Chapter 4, verse 119 as proof that cultivated meat is nothing but a pointless experiment with nature [37,70,71]. However, this argument can be criticised because the exact meaning of “altering God's creation” in this verse is to alter without a clear purpose and the possibility to harm human beings [30]. Hossain further contends that cultivated meat production is unnatural and not in line with the *tayyiban* concept [37]. Nonetheless, his argument may not be universally applicable, given the application of genetic modification in food research and industry. In his opinion, all animal and plant-based products produced with the help of genetic engineering are unnatural, but the definition of ‘unnatural’ remains undefined [72].

### 4.4 Fourth: basic principles for halal production of cultivated meat

The majority of Muslim scholars addressing the halal requirements for halal cultivated meat agree on several fundamental principles [9,23,25,27,36,38,[40], [41], [42], [43], [44], [45], [46], [47], [48], [49], [50], [51]]:

i. The source of stem cells must be obtained from halal slaughtered animals such as chickens, cows, goats, fish and shrimps. If there is cross-contamination with other non-halal stem cells, it may result in a non-halal cultivated meat.
ii. The halal animal must be slaughtered according to Islamic teachings.
iii. No non-halal element, substances or components may be employed during the process, for example animal blood serum, non-halal animal-based gelatine, enzymes or growth factors derived from non-halal animals or from halal animals that were still alive during extraction.
iv. The utilities and storage space used during the cultivated meat production process must be separated from halal and non-halal elements to avoid cross-contamination.
v. No harm is brought to any human beings.

### 4.5 Fifth: The necessity of cultivated meat in the present world situation from the perspective of al-maqasid al-shariah

The perspective of *al-maqasid al-shariah*, which encompasses the higher objectives of Islamic rulings, underscores the importance of preserving the five primary elements, i.e. the preservation of religion, life, property, intellect and lineage, without neglecting other elements such as the preservation of the environment, food security and food safety. The cultivated meat production is in line with several elements of *al-maqasid al-shariah*, for example, reducing pollution and ensuring food security in certain countries. The benefits that cultivated meat production brings to people and the environment are referred to as *maslahah* (benefit) in the perspective of *al-maqasid al-shariah*. Some scholars classify the consumption of cultivated meat into three levels of necessity for the preservation of human life, namely *daruriyyat* (the essential), *hajiyyat* (the supplementary) and *tahsiniyyat* (the desirable or embellishment) [9,51]. Applying this analytical framework, certain countries may view cultivated meat production as reaching the level of *daruriyyat* (the essential) due to constraints such as limited land availability, which jeopardises their food security. One example is Singapore which has been very active in the alternative protein space and is the first nation to approve the commercial sale of cultivated meat [73,74].

## 5 Conclusion

In conclusion, the emergence of cultivated meat in the food industry poses an important problem in terms of its halal status. Muslim consumers adhere to dietary principles that require meat to meet both halal and *tayyib* standards. To determine the halal status of cultivated meat, several important considerations must be made:

(i) The ‘life character’ of cultivated meat, which reflects the Islamic view of the creation of life in this new context.
(ii) The status and origin of the stem cells used in the culture process to ensure that they are derived from halal sources.
(iii) The implications of cultivated meat production for altering God's creation are consistent with the theological concerns surrounding alteration.
(iv) Basic principles for the production of halal cultivated meat, covering the entire process from sourcing to consumption.
(v) The need for cultivated meat in the contemporary world from an *al-maqasid al-shariah* perspective, while exploring the broader social and ethical implications.

If cultivated meat is to be considered halal and serve the large Muslim consumer market, these aspects need to be carefully addressed. It is recommended that established fatwa bodies, including the International Islamic Fiqh Academy (IIFA), collaborate and take immediate action to establish clear guidelines and standards for cultivated meat. Their rulings are of great importance to the Muslim world and their involvement is essential to harmonise the handling of halal cultivated meat in all regions. Key players in the halal business and welfare sectors such as JAKIM (Department of Islamic Development Malaysia) and MUIS (The Office of the Mufti of Singapore) are encouraged to take the lead in this initiative as they recognise the increasing importance of cultivated meat in the food industry. These joint efforts can ensure that cultivated meat meets halal and *tayyib* standards and fulfil the needs and expectations of Muslim consumers on a global scale [75].

## Ethics statement

Review and/or approval by an ethics committee was not needed for this study because this study does not involve human participants or animal experimentation.

## Credit authorhip contribution statement

**Mohammad Naqib Hamdan:** Writing – original draft, Methodology, Funding acquisition, Formal analysis, Conceptualization. **Rufaihah Abdul Jalil:** Supervision, Data curation, Conceptualization. **Mohd Anuar Ramli:** Supervision, Methodology. **Nasiibah Ramli:** Formal analysis, Data curation, Conceptualization. **Mohd Nor Adzhar Ibrahim:** Validation, Methodology. **Muhamad Firdaus Ab Rahman:** Writing – review & editing, Supervision. **Hussein ‘Azeemi Abdullah Thaidi:** Writing – review & editing, Supervision, Methodology. **Nur Najwa Hanani Abd Rahman:** Supervision, Methodology, Data curation.

## Declaration of competing interest

Mohammad Naqib Hamdan reports financial support was provided by 10.13039/501100005417Universiti Teknologi Malaysia.
